# Gramado consensus on imaging evaluation of the response to neoadjuvant systemic therapy for breast cancer

**DOI:** 10.1590/0100-3984.2025.0101

**Published:** 2026-07-08

**Authors:** Linei Augusta Brolini Delle Urban, Luciano Fernandes Chala, Ivie Braga de Paula, Beatriz Medicis Maranhão Miranda, Almir Galvão Vieira Bitencourt, Paula de Camargo Moraes, Ana Claudia Mendes Rodrigues Mussauer, Carla Cristina Teixeira Polimeni Benetti, Ana Claudia Silveira Racy, Lucio De Carli, Bruna Maria Thompson, Henrique Lima Couto, Eduardo Carvalho Pessoa, Nilceana Maya Aires Freitas, Daniel Fontes Santos de Teive e Argolo, Filomena Marino Carvalho

**Affiliations:** 1 Clínica de Diagnóstico Avançado por Imagem (DAPI), Curitiba, PR, Brazil; 2 Grupo Fleury Medicina e Saúde, São Paulo, SP, Brazil; 3 Instituto Orizonti de Longevidade e Saúde, Belo Horizonte, MG, Brazil; 4 Lucilo Maranhão Diagnósticos, Recife, PE, Brazil; 5 A.C.Camargo Câncer Center, São Paulo, SP, Brazil; 6 Grupo Dasa, São Paulo, SP, Brazil; 7 Hospital São Vicente de Paulo, Rio de Janeiro, RJ, Brazil; 8 Beneficência Portuguesa de São Paulo, São Paulo, SP, Brazil; 9 Hospital Israelita Albert Einstein, São Paulo, SP, Brazil; 10 Hospital Mãe de Deus, Porto Alegre, RS, Brazil; 11 Hospital Nora Teixeira, Porto Alegre, RS, Brazil; 12 University of Iowa Hospitals and Clinics, Iowa City, IA, USA; 13 Redimama-Redimasto, Belo Horizonte, MG, Brazil; 14 Universidade Estadual Paulista, UNESP, Botucatu, SP, Brazil; 15 Hospital de Câncer Araújo Jorge, Goiana, GO, Brazil; 16 Cebrom Oncoclínicas, Goiana, GO, Brasil; 17 Instituto de Mastologia e Oncologia, Goiana, GO, Brazil; 18 Grupo Oncoclínicas, Salvador, BA, Brazil; 19 Faculdade de Medicina da Universidade de São Paulo (FMUSP), São Paulo, SP, Brazil

**Keywords:** breast neoplasm, neoadjuvant systemic therapy, imaging assessment, breast magnetic resonance imaging;

## Abstract

The assessment of response to neoadjuvant systemic therapy (NST) is a critical pillar in defining the multidisciplinary therapeutic strategy in breast cancer. This consensus aimed to establish national guidelines for imaging assessment of NST, based on the best scientific evidence adapted to the Brazilian context. To this end, a panel of experts was formed, composed of breast radiologists, breast surgeons, oncologists, onco-radiotherapists and breast patholo-gists. The methodology was based on a systematic literature review, followed by face-to-face discussions and rounds of virtual voting (adapted Delphi method), defining consensus when agreement was =75%. As a result, radiological response criteria were standardized, and accuracy, false-negative, and positive rates were determined, as well as the role of mammography, ultrasound, magnetic resonance imaging, and tomosynthesis, stratified by molecular subtypes. Additionally, recommendations were established for marking the breast lesion and axillary lymph nodes before the start of treatment. Finally, the role of other imaging techniques, such as contrast-enhanced mammography and pos-itron-emission tomography/computed tomography, was discussed, as well as the impact of artificial intelligence as tools for evaluating the response to SNRT.

## INTRODUCTION

Neoadjuvant systemic therapy (NST) has become established as an essential therapeutic strategy in the management of breast cancer. It can reduce the extent of breast and axillary surgery, expanding the possibilities for breast preservation, as well as allowing *in vivo* evaluation of the tumor response to treatment and providing valu-able prognostic information. Accurate evaluation of the response to NST is indispensable for future therapeutic decisions, including the extent of surgical resection and adjuvant treatment. In this scenario, imaging methods play a central role in estimating the extent of residual tumor, in the breast and in the axillary lymph nodes^**(^1^)**^. However, heterogeneity in the techniques employed and in the criteria applied for interpretation and classification, as well as in the experience of the professionals involved, can generate significant variations in the results and con-sequent clinical impact^**(^2^)**^. Faced with these challenges, specialized multidisciplinary debate and the development of guidelines are essential to guide imaging assessment in this clinical context. The objective of this article is to report the recommendations of a multidisciplinary panel of experts convened to discuss and establish a consensus on the critical steps related to the use of imaging meth-ods in the evaluation of the response to NST for breast cancer in Brazil. Future directions for the application of artificial intelligence (AI) and the development of new imaging techniques are also discussed.

### Search of the literature

**Figure 1:**
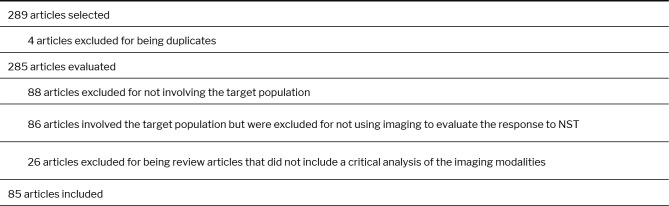
Flow chart of the article selection process.

To support the discussions, a systematic search of the literature was conducted in the databases Medline (via PubMed) and Latin-American and Caribbean Health Sciences Literature (via the Brazilian Regional Library of Medicine), using descriptors and National Library of Medicine Medical Subject Headings related to the theme (neoadjuvant chemotherapy, diagnostic imaging, and breast neoplasms). Studies, including clinical trials, systematic re-views/meta-analyses, randomized clinical trials, and review articles, were considered eligible if they were published between July 2014 and July 2024, in Portuguese or Eng-lish. Studies were selected if they addressed the imaging evaluation of the response to NST for breast cancer using methods such as mammography, ultrasound, magnetic reso-nance imaging (MRI), tomosynthesis, contrast-enhanced mammography (CEM), and positron-emission tomogra-phy/computed tomography (PET/CT). After those search criteria had been applied, 289 references were identified. Two reviewers, working independently, selected the stud-ies using Rayyan software (Qatar Computing Research Institute, Doha, Qatar). In cases of disagreement, the final decision was made by a third reviewer. Articles that did not address the target population or the scope of the panel were excluded, as were articles of low quality or low methodological rigor. Of the 289 articles initially selected, 204 were thus excluded. Therefore, the final sample com-prised 85 articles selected to support the development of the panel recommendations (Figure 1).

## METHODOLOGY

### Composition of the expert panel

A multidisciplinary panel was convened. The panel was composed of 16 specialists with recog-nized expertise in the fields of breast radiology, breast surgeons, clinical oncology, radiotherapy, and breast pathology.

### Development of the themes and questions

The panel defined 10 themes to be addressed during the in-person meeting, covering the radiological aspects of the response to NST in breast cancer (Chart 1). For each theme, questions were formulated to be answered and put to a vote. Each specialist had 40 days to propose a preliminary text for each question based on the best available scientific evidence.

**Chart 1:** The ten themes addressed by the consensus panel.

1) Pattern of response to NST in the breast and axilla
2) Indications for imaging examinations before, during, and after NST
3) Accuracy of imaging examinations in evaluating the response to NST
4) Management of tumors with calcifications detected on mammography
5) MRI protocol for evaluating the response to NST
6) Use of DWI in evaluating the response to NST
7) Standardization of the measurement of radiological partial response by MRI
8) Standardization of the evaluation of the radiological complete response by MRI
9) Standardization for disease progression through imaging during NST
10) Future directions for image-based evaluation of the response to NST

### Consensus meeting

An in-person consensus meeting took place during the 18th Gramado Breast Cancer Event, in the city of Gramado, located in the Brazilian state of Rio Grande do Sul, and was open to the public. Each panelist was given 7-10 min to present the evidence relevant to their clinical question. Next, an open vote was held for each question with the following response options: “agree”, “disagree”, and “abstain”. Consensus was considered to have been reached when ≥ 75% of the panelists agreed with the statement(s) presented. If that proportion was not achieved, the question was submitted to new rounds of discussion, reformulation, and voting, carried out in person (on the day of the meet-ing), virtually (later), or both, until consensus was reached on all questions proposed by the group.

### Validation and final approval of the recommendations

**Figure 2:**
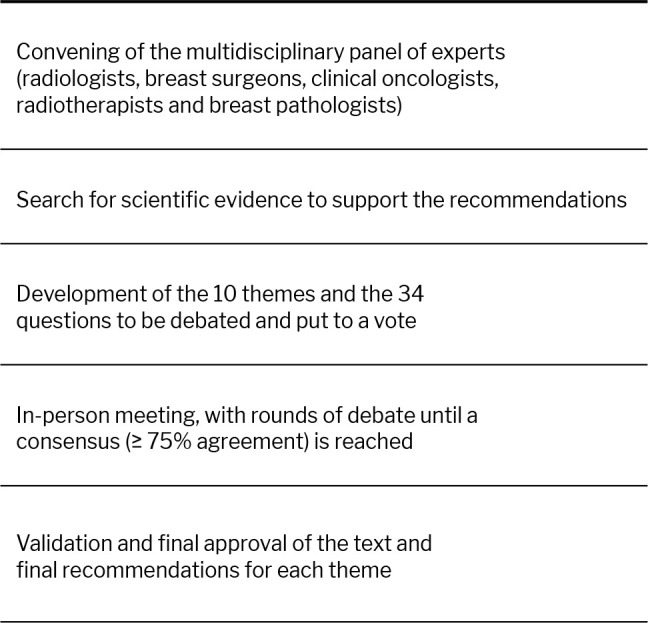
Stages for building the consensus.

After consensus was reached, the text was drafted in a preliminary format and submitted for critical analysis and approval by the panel members. The final recom-mendations were then classified into one of five cat-egories: category A-strong recommendation in favor, based on high-quality evidence; category B-strong recommendation in favor, based on moderate-quality evidence; category C-weak recommendation in favor, based on low-quality evidence; category D-recommen-dation in favor, based solely on an expert consensus; and category E-recommendation against, with insuf-ficient evidence to support its use. The phases of the process are summarized in Figure 2.

## RESULTS

The ten themes proposed for discussion by the panel are listed in Chart 1. For each theme, three to five questions were formulated; a total of 36 questions were put to a vote. Among those 36 questions, consensus was achieved in the first in-person vote for 25 (69.4%), in the first round of virtual voting for two (5.5%), in the second round of virtual voting for three (8.3%), in the third round of virtual voting for three (8.3%), and in the final round of virtual voting for three (8.3%). The answers to the questions are described below, together with the results of the voting by the expert panel. Ultimately, 10 recommendations were issued for the imaging-based evaluation of the response to NST.

## THEME 1: PATTERN OF RESPONSE TO NST IN THE BREAST AND AXILLA

### What types of radiological responses can be identified in the breasts?

**Figure 3:**
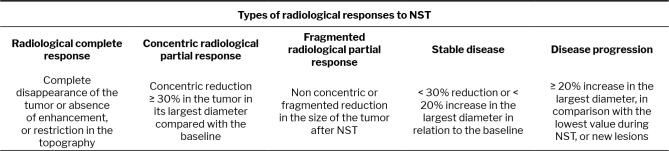
Types of radiological responses to NST.

The types of responses observed in breast imaging examinations are categorized as follows (Figure 3): a radiological complete response-defined as complete disappearance of the tumor (in morphology-based ex-aminations, such as mammography, tomosynthesis, and ultrasound) or the absence of enhancement or restriction in the topography of the tumor (in functional examina-tions, such as MRI, PET/CT, and CEM); a concentric radiological partial response-defined as a concentric reduction ≥ 30% in the size of the tumor in its largest diameter compared with the initial examination; a fragmented radiological partial response-defined as not concentric or fragmented reduction in the size of the tumor after NST; stable disease-defined as a < 30% reduction or a < 20% increase in the largest diameter of the tumor in relation to the initial examination; and disease progres-sion-defined as a ≥ 20% increase in the largest diameter of the lesion, in comparison with the lowest value recorded since the beginning of therapy, or as the appearance of new lesions. These response patterns affect the accuracy of imaging examinations in identifying residual tumor, with the fragmented response pattern having the lowest agreement with the pathology^**(^3^,^4^)**^.

***Panel results: 88% agreement; 0% disagreement; 12% abstention***.

### How can axillary lymph nodes be evaluated and described to allow pretreatment determination of the tumor burden?

**Figure 4:**
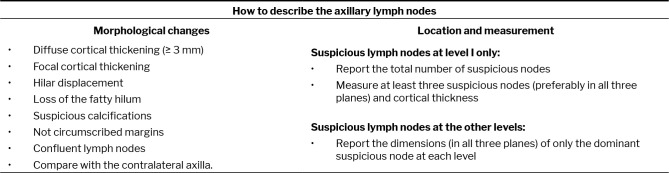
How to describe the axillary lymph nodes.

The ultrasound assessment should be performed with the patient in the supine position, with one hand behind the head. The scan is initiated from the axillary tail of the breast toward level I of the axilla, proceeding to levels II and III^**(^5^)**^. Given that ultrasound assessment plays a role in therapeutic decision-making^**(^6^,^7^)**^, it is extremely important to report the type of morpho-logical alteration observed, as well as the respective level at which the involvement is located (Figure 4).

The following morphological signs are suggestive of metastatic involvement: diffuse cortical thickening (≥ 3 mm); focal cortical thickening; hilar displacement; loss of the fatty hilum; calcifications with suspicious morphology; ill-defined margins; confluent lymph nodes. It is important to compare with the contra-lateral axilla, mainly to evaluate the initial signs of involvement. When there are suspicious lymph nodes only at axillary level I, it is recommended that the total number of suspicious lymph nodes be reported, that the size of at least three such lymph nodes be mea-sured (preferably in all three orthogonal planes), and that any cortical thickening be described. If there are suspicious lymph nodes at the other levels (levels II and III), the recommendation is to describe only the dominant suspicious lymph node at each level, mea-suring its size in all three planes. Doppler evaluation is optional and can be useful to assist in performing the biopsy by identifying the axillary vessels or large-caliber vessels adjacent to the lymph node.

***Panel results: 88% agreement; 0% disagreement; 12% abstention***.

### Is it recommended to use the Response Evalua-tion Criteria in Solid Tumors method for evaluating the response to NST on imaging?

The Response Evaluation Criteria in Solid Tumors (RECIST) method was developed to standardize the evaluation of the response of solid tumors to cancer treatment, being widely used in clinical research and, in some cases, clinical practice. It was published in 2000 and updated (to version 1.1) in 2009. It provides guidance on how to measure and classify target lesions, through imaging examinations, as well as on how to determine whether those lesions have regressed, remained stable, or progressed after treatment. The RECIST method is rarely used or recommended in patients with breast cancer receiving NST, because it presents significant limitations for practical application. Although some of the param-eters established in the RECIST method were used for the development of this consensus, its use in isolation for response evaluation is not recommended. Therefore, we recommend instead using the largest tumor diameter, comparing it with that obtained in the initial examina-tion, as a parameter for evaluating the response to NST, as well as volume and diffusion as optional parameters.

***Panel results: 88% agreement; 0% disagreement; 12% abstention***.

## THEME 2: INDICATIONS FOR IMAGING EXAMINATIONS BEFORE, DURING, AND AFTER NST

### Which examinations are important in the imaging evaluation of the response to NST?

**Figure 5:**
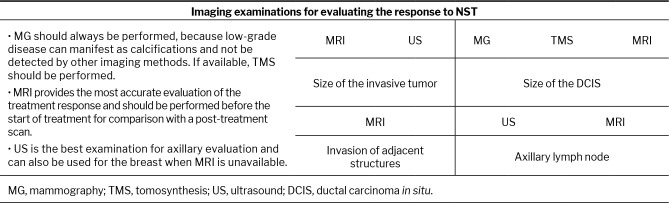
Imaging examinations for evaluating the response to NST.

In the evaluation of the response to NST, mam-mography should always be performed, because low-grade disease can manifest as calcifications and not be detected by other methods, such as MRI. If available, tomosynthesis should be performed, especially in women under 50 years of age with dense breasts and tumors that present with architectural distortion or asymmetry. The examination that provides the most information about the disease is MRI, which, if available, should be performed prior to the start of treatment and again, for comparison, during or at the end of treatment. The best imaging method for axillary evaluation is ultrasound, which can be used in order to evaluate a breast neoplasm when MRI is not available (Figure 5).

***Panel results: 97% agreement; 3% disagreement; 0% abstention***.

### When and for what purposes should these examinations be performed?

**Figure 6:**
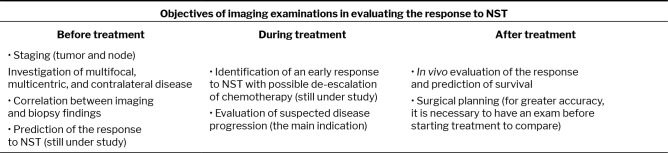
Objectives of imaging examinations in evaluating the response to NST.

The objectives of and indications for imaging methods in NST vary according to the stage of treatment (Figure 6). The objectives before the start of treatment are as follows: to identify and assess the extent of disease in the breast and axilla, including the size of the index lesion and the presence of multifocal/multicentric disease or disease in the contralateral breast; to establish a basis for future comparisons; to correlate imaging aspects with biopsy results in order to exclude cases of diagnostic underestimation or sampling error before the start of treatment; and to identify radiological findings that are predictive of a treatment response (this use is an area of intense research). The following are the objectives during treatment: to identify an early tumor response to NST with possible de-escalation of chemotherapy (this use has been extensively studied but the evidence is still under construction); and to investigate the suspicion of disease progression (in practice, this is the main indica-tion during treatment, and the examination should be chosen according to the clinical presentation and initial tumor presentation). At the end of treatment, the main objective is to identify and assess the extent of residual disease in the breast and axilla, for surgical planning^**(^8^)**^.

***Panel results: 88% agreement; 3% disagreement; 9% abstention***.

### When is breast lesion marking essential, and what materials should be used?

Invasive breast cancer marking should be performed regardless of molecular subtype, preferably before the start of treatment or after the first cycle of treatment, with a metallic clip or radioactive device (radioactive seed), which can be visualized with different imaging methods after the end of NST, or with a charcoal solution, if the team is familiar with that method (which requires visu-alization by a surgeon during surgery, with no possibility of preoperative marking by the radiologist after the end of NST). In patients with a solitary lesion, the marker should be inserted in the center of the lesion. If there are multiple lesions (multifocal, multicentric, or both), those that influence the surgical planning should be marked. Bilateral lesions should always be marked^**(^9^)**^.

***Panel results: 90% agreement; 0% disagreement; 10% abstention***.

### When is axillary lymph node marking essential, and what material should be used?

Despite advances in imaging, no imaging examina-tion reliably rules out axillary involvement (any node status), with surgical biopsy being the gold standard for determining axillary response. However, the concept of axillary tumor burden determined by imaging methods is replacing the concept of lymph node involvement in the initial staging of breast cancer (low tumor burden generally being defined as fewer than two suspicious lymph nodes on imaging). This concept of residual axil-lary tumor burden has not yet been widely validated for use in evaluating the axillary response after NST. The aim of marking the affected lymph node before the start or after the first cycle of NST (with a clip, radioactive device, or charcoal) is to prepare for targeted axillary dissection, which reduces the false-negative rate of axillary staging^**(^10^)**^. Nevertheless, its implementation and technique are under debate and should follow the protocols of the respective institution.

***Panel results: 90% agreement; 0% disagreement; 10% abstention***.

## THEME 3: ACCURACY OF IMAGING EXAMINATIONS IN EVALUATING THE RESPONSE TO NST

### Does the accuracy of imaging examinations vary according to tumor subtype?

Imaging examinations play a key role in evaluating the response to NST, assisting in surgical planning and avoiding overtreatment. Accuracy can vary depending on the imaging method and the molecular subtype of the tumor, being lower for luminal tumors and higher for triple-negative tumors, as well as for those with HER2 overexpression^**(^11^)**^.

***Panel results: 100% agreement; 0% disagreement; 0% abstention***.

### How accurate is mammography in evaluating the response to NST?

In mammography, lesion measurement shows a weak correlation with pathology, with an average coef-ficient of agreement of 0.55^**(^11^)**^. For the identification of a pathological complete response (pCR), mammography has been reported to have a sensitivity of 65%, a speci-ficity of 81%, a positive predictive value (PPV) of 52%, a negative predictive value (NPV) of 88%, and a rate of agreement with the pathological evaluation of 40%^**(^12^)**^.

***Panel results: 100% agreement; 0% disagreement; 0% abstention***.

### How accurate is ultrasound in evaluating the response to NST?

Ultrasound is an effective technique when there is residual tumor with a diameter ≥ 0.7 cm. However, its reported accuracy in predicting a response to treatment is only 59.6-80.0%, being limited when the lesion is a ductal carcinoma *in situ*, for which it has a sensitivity of 60-80%, a specificity of 70-85%, a PPV of 65-75%, and an NPV of 65-75%^**(^13^)**^.

***Panel results: 88% agreement; 12% disagreement; 0% abstention***.

### How accurate is MRI in evaluating the response to NST?

In comparison with mammography and ultrasound, MRI has the best accuracy, being the modality recom-mended as the gold standard for evaluating the response to NST^**(^14^)**^. It has a sensitivity of approximately 90%, a specificity of 60-100%, a PPV of 65-85%, and an NPV of 85-90%. The accuracy of MRI is higher for HER2-positive tumors, and triple-negative tumors, whereas it is lower for luminal subtypes A and B^**(^15^)**^. It has also shown better accuracy in predicting a pCR for tumors with a high proliferative index.

***Panel results: 82% agreement; 0% disagreement; 18% abstention***.

## THEME 4: MANAGEMENT OF TUMORS WITH CALCIFICATIONS DETECTED ON MAMMOGRAPHY

### What types of responses to NST can calcifications exhibit?

The type of tumor regression and treatment-induced changes affect the accuracy of imaging methods in identi-fying and determining the extent of residual disease after NST. Although the ability of mammography to detect calcifications is an advantage in breast cancer screening, it can become a disadvantage after treatment. Calcifications can increase, decrease, or remain stable after NST^**(^16^)**^: in most cases (50-83%), they remain stable; in 12-18% of cases, they decrease, disappearing completely in only 4%; and they increase in 8-29% of cases.

***Panel results: 100% agreement; 0% disagreement; 0% abstention***.

### What does the persistence of calcifications after the end of NST represent?

Calcifications may represent residual tumor (in-vasive or ductal carcinoma *in situ*), tumor necrosis, or other, treatment-related changes. From their morphol-ogy, extent, or evolution on mammography, it is not possible to establish whether they represent residual tumor or benign changes. Studies have shown that 50.4-61.5% of post-treatment calcifications at the tumor site represent residual tumor, and a number of studies have reported a poor correlation between their extent on mammography and the dimensions of the tumor in the surgical specimen^**(^12^,^17^)**^.

***Panel results: 100% agreement; 0% disagreement; 0% abstention***.

### How should we manage residual calcifications seen on mammography that do not show enhancement on MRI?

A negative result on MRI does not have sufficient accuracy to establish the absence of residual tumor in the clinical scenario of calcifications identified on mam-mography^**(^18^,^19^)**^. Thompson et al.^**(^18^)**^ found that residual tumor was present in 76% of women with post-NST cal-cifications on mammography and a radiological complete response (absence of any type of enhancement) on MRI. Therefore, complete excision of post-NST calcifications in the tumor bed is recommended, regardless of their mam-mographic appearance, even if there is complete nodule regression and absence of enhancement on MRI^**(^19^)**^. This ensures the resection of any residual tumor and facilitates future surveillance. However, it represents a limitation to post-NST surgical de-escalation, potentially resulting in unnecessary mastectomies in many women.

***Panel results: 100% agreement; 0% disagreement; 0% abstention***.

## THEME 5: MRI PROTOCOL FOR EVALUATING THE RESPONSE TO NST

### Should the full MRI protocol always be used, or can the abbreviated version be applied?

The full MRI protocol is the most suitable for evalu-ating the response to NST, given that there are no robust data in the literature to support the use of an abbreviated protocol^**(^20^)**^. The relevant studies have been retrospective, with small samples and without standardized protocols, which can mainly result in underestimation of residual tumor requiring surgical re-intervention^**(^20^,^21^)**^.

***Panel results: 100% agreement; 0% disagreement; 0% abstention***.

### At what stage should MRI be performed and for what purposes?

It is recommended that MRI be performed before the start of NST (as a baseline examination) to assess the extent of the disease and the presence of multifocal, multicentric, or bilateral lesions. During treatment, a follow-up MRI may be performed after completion of the first or second cycle to evaluate the response to treatment, given the possibility of altering the treatment regimen when it is not effective, although this is still under study and currently without clinical application. After the end of treatment, MRI assists in surgical planning, especially when conservative techniques are employed, precluding the need for a more extensive surgical procedure and reducing the need for reoperation due to residual disease or positive margins.

***Panel results: 82% agreement; 0% disagreement; 18% abstention***.

### When is it necessary to proceed with a biopsy in the presence of a new suspicious finding on MRI prior to treatment?

Suspicious lesions, with an appearance different from the index lesion (suspected of being other tumor subtypes), and those that might modify the treatment plan should be biopsied prior to the start of the pro-posed treatment regimen.

***Panel results: 100% agreement; 0% disagreement; 0% abstention***.

### What should be done if an MRI finding is probably benign before the start of treatment?

Category 3 of the Breast Imaging Reporting and Data System (BI-RADS) is applied to findings consid-ered “probably benign,” with a less than 2% chance of malignancy. Although BI-RADS category 3 findings are well-defined and have been validated for mammography and ultrasound, there are still limited data for MRI^**(^22^)**^. Although the use of category 3 as a criterion is discour-aged in the context of imaging evaluation of a known neoplasm, lesions of that character are often found, and their management is challenging. Because the patient is already scheduled for treatment and has a high risk of malignancy, some professionals choose to confirm these lesions by biopsy, especially if the lesions in question are near the index lesion. However, several studies have demonstrated a low (0.8-1.4%) risk of malignancy for patients with BI-RADS 3 lesions^**(^23^,^24^)**^, which could justify imaging follow-up. Therefore, the best course of action should be individualized and discussed in a multidisci-plinary context, taking into account the type of lesion, the risk group, and the treatment proposed.

***Panel results: 94% agreement; 0% disagreement; 6% abstention***.

## THEME 6: USE OF DIFFUSION-WEIGHTED IMAGING IN EVALUATING THE RESPONSE TO NST

### What are the parameters for a good diffusion-weighted imaging sequence?

Diffusion-weighted imaging (DWI) can be used in order to assess the response to NST. These sequences do not require the use of contrast and allow quantita-tive assessment through calculation of the apparent diffusion coefficient (ADC). The necessary equipment, parameters, and cutoff point that allow good differen-tiation between benign and malignant lesions on DWI have been well established in the recommendations of the European Society of Breast Imaging^**(^25^-^27^)**^, covering several aspects: a ≥ 1.5 T scanner; a dedicated coil with at least 4 channels; axial acquisitions including both breasts; acquisition preferably before contrast admin-istration; an echo-planar imaging spectral attenuated inversion recovery sequence with fat saturation; minimum resolution of 2 × 2 mm; slice thickness ≤ 4 mm; the use of two b values (0-50 s/mm^2^ and 800 s/mm^2^); and an acquisition time of 2-5 min. However, the low resolution and long acquisition time are factors that still limit the use of DWI at many institutions.

***Panel results: 75% agreement; 0% disagreement; 25% abstention***.

### What values are used in order to determine the response to NST?

Several studies and meta-analyses have sought to establish ADC cutoff values to differentiate patients who have achieved a pCR, as well as to predict, at the beginning of treatment, those with a higher probability of achieving a pCR ^**(^27^)**^ . However, there is considerable heterogeneity among the published works, in relation to the population studied, the type of treatment used, the DWI acquisition technique, and the post-processing of the images. This variability makes it difficult to define universally applicable ADC cutoff values. However, tu-mors with lower ADC values before the start of therapy tend to show a better response to treatment and a greater chance of achieving a pCR. In addition, an increase in ADC over the course of treatment generally reflects a tumor response. In the absence of exact cutoff values, experts recommend that, for early response assessment (after the first or second cycle of treatment), the criterion of a > 25% increase in the ADC values over the baseline values should be used in order to identify patients who have achieved a good response^**(^28^,^29^)**^. For the end-of-treatment analysis, a documented increase in the ADC values (> 40% over baseline) suggests a radiological complete response, as do ADC values similar to or above those observed for normal breast tissue^**(^30^)**^. Conversely, the persistence of low ADC values, close to those ob-served before treatment, suggests residual tumor.

***Panel results: 75% agreement; 0% disagreement; 25% abstention***.

### How accurate is DWI in evaluating the response to NST?

In the assessment of the early response to NST (after the first or second cycle of treatment), a > 25% increase in the ADC has been shown to have a sensitivity of 83%, a specificity of 76-84%, and an accuracy of 84% for iden-tifying a pCR^**(^28^,^29^)**^, whereas an ADC increase > 40% has been shown to have a sensitivity of 100%, a specificity of 91%, and an accuracy of 96% for identifying a pCR at the end of treatment^**(^30^)**^. A recent meta-analysis of 21 articles demonstrated that, for identifying a pCR,/ the use of DWI has a sensitivity of 89% (range, 86-91%) and a specificity of 72% (range, 68-75%) in patients undergoing NST^**(^31^)**^.

***Panel results: 75% agreement; 0% disagreement; 25% abstention***.

## THEME 7: STANDARDIZATION OF THE MEASUREMENT OF A RADIOLOGICAL PARTIAL RESPONSE BY MRI

### What are the methods for quantifying a partial response, depending on the type of lesion?

The method for quantifying a partial response de-pends on the observed pattern of response to NST. The measurement is most reproducible in cases of a concentric partial response, in which there is a pattern of retraction or shrinkage, with the best correlation being between the size of the lesion on imaging and the pathology result. In this measurement, the entire lesion should be included in all three planes with multiplanar reconstruction (antero-posterior, latero-lateral, and craniocaudal axes) and key images (in at least the two orthogonal planes containing the three major axes) should be obtained for documenta-tion. In the case of a concentric partial response in a lesion containing areas of necrosis, the cystic portions without enhancement should be included in the measurement of the lesion in the three major planes. In the case of a fragmentation response pattern, the total extent of the lesion in all three planes should be measured (to allow surgical planning), as should the largest residual focus, which can be used for staging the lesion, according to the tumor-node-metastasis classification (TNM)^**(^32^,^33^)**^.

***Panel results: 94% agreement; 0% disagreement; 6% abstention***.

### How to quantify a partial response in single, multifocal, multicentric, and bilateral lesions

**Figure 7:**
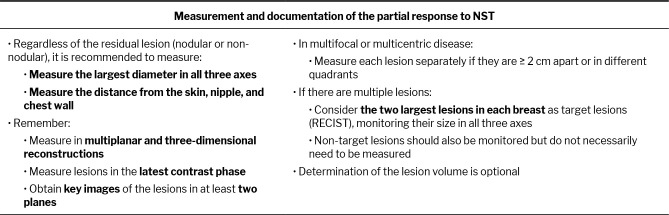
Measurement and documentation of the partial response to NST.

Regardless of the type of residual lesion (nodular or non-nodular), when the lesion is solitary, measure the largest diameter in all three axes, as well as the distance from the skin, nipple, and chest wall. Use multiplanar and three-dimensional reconstructions for this measure-ment, as well as the latest contrast phase (or the phase with the greatest tumor enhancement). Obtain key images of the largest axis in each plane, and document the size in at least two orthogonal planes. In the case of lesions that are multifocal or multicentric, measure each lesion separately, especially if there is a ≥ 2 cm distance between them or if they are in different quadrants. When there are multiple lesions, consider the two largest in each breast as the target lesions (as recommended in the RECIST), monitoring the measurements in all three axes. The re-maining lesions (i.e., the non-target lesions) should also be monitored but do not necessarily need to be measured. The determination of lesion volume is optional but can be useful in some cases (Figure 7).

***Panel results: 81% agreement; 0% disagreement; 19% abstention***.

### What are the most common causes of error in determining tumor size?

The causes of divergence between imaging mea-surements and pathology include the presence of ductal carcinoma *in situ*, an adjacent inflammatory process, the presence of necrosis, non-nodular lesions, residual microscopic foci, anti-angiogenic therapy, and late tumor enhancement^**(^32^,^33^)**^. The correlation is stronger for tumors with an initial diameter ≤ 5 cm, high-grade carcinomas, and hormone receptor-negative tumors.

***Panel results: 94% agreement; 0% disagreement; 6% abstention***.

### How do benign lesions behave over the course of the treatment?

Benign lesions can undergo changes during neoad-juvant chemotherapy, because chemotherapeutic drugs affect vascularization and reduce circulating hormone levels^**(^33^,^34^)**^. The possible effects on benign lesions in-clude the following: none (stability); a reduction in size; a reduction in enhancement, reflecting decreased blood supply or cellular activity; and resolution, especially if the lesions are hormone-sensitive or inflammatory nature.

***Panel results: 94% agreement; 0% disagreement; 6% abstention***.

## THEME 8: STANDARDIZATION OF THE EVALUATION OF THE RADIOLOGICAL COMPLETE RESPONSE BY MRI

### What is the definition of a radiological complete response and the most accurate parameters to identify it: absence of enhancement or enhancement smaller than parenchyma?

It is recommended to adopt the absence of enhance-ment in the later phases of a dynamic MRI study as a criterion for a radiological complete response. This cri-terion is the most widely used in the literature because it has been well studied, has been widely validated, and is easy to implement. In the presence of minimal residual enhancement, the calculation of the signal enhancement ratio (SER) can be useful, given that the parameters with the highest specificity (90.4%) for a pCR are a SER ≤ 1.6 and enhancement extending ≤ 0.2 cm^**(^35^-^37^)**^.

***Panel results: 82% agreement; 6% disagreement; 12% abstention***.

### How should we proceed in cases of lesions in which high signal intensity persists on T2-weighted imaging but there is no enhancement?

Tumors that become necrotic, hemorrhagic, or fibrotic after NST can show high signal intensity on T2-weighted imaging. However, that finding should not necessarily be interpreted as residual lesion, especially if there is no accompanying enhancement^**(^35^-^37^)**^.

***Panel results: 88% agreement; 0% disagreement; 12% abstention***.

### What are the causes of false positives and false negatives in determining a radiological complete response?

The causes of false positives in determining a ra-diological complete response include fibrosis and post-treatment inflammatory changes, which can present as enhancement in the tumor bed. False negatives are mainly related to non-nodular enhancement-type tumors and the antiangiogenic action of chemotherapeutic agents such as taxanes and anthracyclines, which reduce blood flow and vascular permeability^**(^33^,^35^-^37^)**^.

***Panel results: 88% agreement; 6% disagreement; 6% abstention***.

### How to evaluate signs of a radiological complete response in the axilla by MRI?

The response to NST in the axilla has been de-scribed as a return to the usual appearance of the lymph nodes; that is, symmetrical (in relation to the contralateral axilla), with a thin cortex and a preserved fatty hilum, and showing homogeneous contrast enhancement^**(^35^-^37^)**^.

***Panel results: 82% agreement; 0% disagreement; 18% abstention***.

### How accurate are image-guided biopsy methods for confirming a pCR?

Image-guided minimally invasive biopsies have shown promise in identifying a pCR. Although there is no standardization in the literature, it is recommended, on the basis of the best results obtained, that sampling be done by vacuum-assisted biopsy with 7-10 gauge needles, collecting 6-12 representative fragments from the tumor bed, with clip retrieval, in patients who meet the criteria for a radiological complete response or mini-mal residual disease on MRI^**(^37^,^38^)**^.

***Panel results: 88% agreement; 0% disagreement; 6% abstention***.

## THEME 9: STANDARDIZATION OF SCREENING FOR DISEASE PROGRESSION THROUGH IMAGING DURING NST

### What should be the definition of tumor progression and the parameters used in order to identify it?

**Figure 8:**
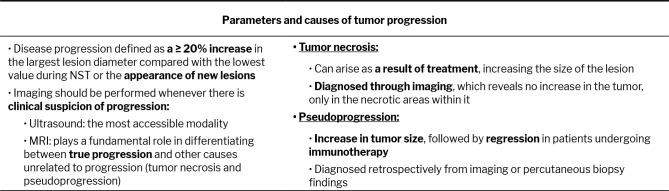
Parameters and causes of tumor progression.

Disease progression is the least common type of response to NST. It is characterized by a ≥ 20% increase in the largest lesion diameter in comparison with the lowest value recorded since the start of therapy or the appearance of new lesions. Early recognition of progression can modify treatment and avoid unneces-sary toxicities. Whenever there is clinical suspicion of progression, imaging studies should be ordered. Ultrasound can be an accessible alternative when it can clearly confirm disease progression. However, in many cases MRI plays a fundamental role in differen-tiating between true progression and other causes of a clinically relevant increase in the size of lesion that are unrelated to growth of the tumor itself, such as tumor necrosis^**(^39^)**^, as described in Figure 8.

***Panel results: 94% agreement; 0% disagreement; 6% abstention***.

### Does the average time to a response after the start of therapy vary according to the molecular subtype?

Triple-negative molecular subtype and tumors with HER2 overexpression that show an early response (after the first cycle of treatment) have a higher probability of achieving a pCR at the end of treatment. Changes in the enhancement curve and diffusion precede changes in dimensions. Conversely, receptor-positive (luminal) tumors undergoing NST have a later response and a lower chance of achieving a pCR^**(^39^)**^.

***Panel results: 94% agreement; 0% disagreement; 6% abstention.***

### What are the factors that can mimic disease progression during NST?

During NST, tumor necrosis and pseudoprogression can mimic progression of breast cancer (Figure 8). Tumor necrosis can arise as a result of treatment, causing an increase in the clinical dimensions of the lesion. The diagnosis is made through imaging examinations, which can confirm that the increase in size is not due an increase in the tumor but rather to expansion of the necrotic areas within it that lead to an overall increase in the lesion size. Pseudoprogression is defined as an increase in tumor size or the appearance of a new lesion followed by regression in patients undergoing immunotherapy. It is diagnosed by using retrospective imaging data. Histopathology shows infiltration of multiple immune cells, rather than tumor cells. Biopsy is a relatively simple procedure that can be used in order to differentiate between true progression and pseudoprogression^**(^40^-^42^)**^.

***Panel results: 94% agreement; % disagreement; 6% abstention***.

## THEME 10: FUTURE DIRECTIONS FOR IMAGE-BASED EVALUATION OF THE RESPONSE TO NST

### How will the introduction of AI change mammography and ultrasound examinations?

The use of AI in combination with imaging methods for predicting the response to NST has been extensively studied in the literature. There have been small, hetero-geneous studies using mammography, some of which have employed AI to evaluate changes in breast density as a means of predicting the response to treatment, with interesting preliminary results^**(^43^)**^. Combining ultrasound with AI algorithms makes it possible to evaluate the bio-physical properties of breast tissue and produce satisfac-tory results in predicting treatment response^**(^44^)**^. Another great potential of AI in ultrasound is the possibility of predicting axillary lymph node metastasis.

***Panel results: 88% agreement; 0% disagreement; 12% abstention***.

### How will the introduction of AI change MRI examinations?

The most accurate method for predicting the treatment response is MRI. Currently, qualitative (morphology and enhancement) data are being com-bined with multiparametric MRI data^**(^4^)**^, as well as radiomics, deep learning, and machine learning data, making this analysis predominantly quantita-tive, with improved accuracy^**(^45^,^46^)**^. The use of AI in combination with the experience of the radiologist and the technological advances in imaging methods has great potential for optimizing the prediction of the response to NST.

***Panel results: 88% agreement; 0% disagreement; 12% abstention***.

### What is the impact of new imaging methods being studied, such as CEM?

The sensitivity of CEM in predicting the treat-ment response is similar to that of MRI. Therefore, CEM represents an acceptable alternative when MRI is contraindicated or unavailable^**(^47^)**^.

***Panel results: 88% agreement; 0% disagreement; 12% abstention***.

### Can PET/CT play a role in evaluating the response to NST?

For predicting the response to NST, PET/CT has greater specificity than does MRI, whereas MRI has greater sensitivity. New radiopharmaceuticals and the combination with radiomics has the potential to improve the performance of PET/CT^**(^9^)**^. Although still in the research phase, dedicated breast PET and PET/ MRI provide better image quality and functional param-eters^**(^2^,^48^)**^, which makes them promising new modalities.

***Panel results: 88% agreement; 0% disagreement; 12% abstention***.

## CONCLUSION

This consensus presents a comprehensive overview of the image-based evaluation of the response to NST, addressing response patterns, examination techniques, measurement methodologies, and documentation of the results, as well as discussing future directions for the application of AI and the development of new imaging techniques (Chart 2). It serves as a practical guide, not only for radiologists but also for all professionals on multidisciplinary teams involved in the care of breast cancer patients, and is consistent with similar documents published in other countries^**(^49^,^50^)**^. However, we recognize that in some regions of Brazil, especially within the setting of the Unified Health Care System, there may be restrictions on access to more complex examinations or procedures, such as MRI or prior marking for NST, which can limit the full applicability of these recommendations at facilities with less infrastructure or of lesser complexity. In such situations, it is essential that each institution follow the established protocols, considering local availability. Finally, we emphasize that this document will be reviewed periodically, with updates planned every three years, thus ensuring the incorporation of new evidence and technologies.

**Chart 2:** Final recommendations for imaging assessment after NST.

Recommendation	Level of evidence
• Knowledge of the tumor molecular subtype and response patterns is recommended for better accuracy in imaging evaluation after NST.	Category C
• It is recommended that mammography be performed before and at the end of NST, because calcifications related to low-grade tumors can be seen only on a mammogram. Consider tomosynthesis whenever available.	Category B
• MRI is recommended as the gold-standard method for evaluating the response to NST, and a baseline examination is essential before the start of treatment.	
• In cases of a radiological partial response, stable disease, or progression, the tumor should be measured in the three major axes and documented in key images (in at least two orthogonal planes); the measurement of tumor volume is optional. In the case of a radiological complete response, the absence of enhancement at the tumor site in later stages is the most widely used criterion. The use of DWI increases the accuracy for predicting a pCR.	Category B
• Ultrasound is the preferred examination for axillary staging; it can also be used to assess the breast lesion response when MRI is unavailable. Because ultrasound evaluation of axillary lymph nodes plays a role in therapeutic decision-making, it is extremely important to report the type of morphological alteration observed, the number of suspicious lymph nodes, and the axillary level at which such lymph nodes are located.	Category B
• It is recommended that breast tumor marking be performed regardless of the molecular subtype, preferably before the start of or after the first cycle of treatment, even for multifocal/ multicentric lesions that modify surgical planning, as well as for bilateral malignant lesions. Axillary lymph node marking is widely debated and should be performed after a multidisciplinary discussion and according to protocols established by the respective institution.	Category D
• At the end of treatment, complete excision of calcifications in the tumor bed is recommended, regardless of their mammographic appearance, even if there is complete nodule regression and absence of enhancement on MRI.	Category C
• Al, combined with the expertise of radiologists and the technological development of imaging methods, has the potential to optimize the prediction of the response to NST.	Category D

Category A-strong recommendation in favor, based on high-quality evidence; Category B-strong recommendation in favor, based on moderate-quality evidence; Category C-weak recommendation in favor, based on low-quality evidence; Category D-recommendation in favor, based solely on an expert consensus; Category E-recommendation against, because the evidence is insufficient to support its use.
